# Variation in Bacterial and Fungal Communities in Soils from Three Major Apple Pear (*Pyrus bretschneideri* Rehd.) Orchards

**DOI:** 10.3390/microorganisms12091751

**Published:** 2024-08-23

**Authors:** Guangze Lyu, Jiayang Hu, Jincai Ma

**Affiliations:** 1 Key Laboratory of Ground Water Resource and Environment, Ministry of Education, Jilin University, Changchun 130021, China; lvgz19@mails.jlu.edu.cn; 2 Jilin Provincial Key Laboratory of Water Resources and Environment, Jilin University, Changchun 130021, China; hjy23@mails.jlu.edu.cn

**Keywords:** bacterial community, fungal community, network analysis, soil, structure equation model, sustainable management

## Abstract

Microbial communities are closely related to the overall health and quality of soil, but studies on microbial ecology in apple pear orchard soils are limited. In the current study, 28 soil samples were collected from three apple pear orchards, and the composition and structure of fungal and bacterial communities were investigated by high-throughput sequencing. The molecular ecological network showed that the keystone taxa of bacterial communities were *Actinobacteria*, *Proteobacteria*, *Gemmatimonadetes*, *Acidobacteria*, *Nitrospirae*, and *Chloroflexi*, and the keystone taxon of fungal communities was *Ascomycota*. Mantel tests showed that soil texture and pH were important factors shaping soil bacterial and fungal communities, and soil water soluble organic carbon (WSOC) and nitrate nitrogen (NO_3_^−^-N) were also closely related to soil bacterial communities. Canonical correspondence analysis (CCA) and variation partition analysis (VPA) revealed that geographic distance, soil texture, pH, and other soil properties could explain 10.55%, 13.5%, and 19.03% of the overall variation in bacterial communities, and 11.61%, 13.03%, and 20.26% of the overall variation in fungal communities, respectively. The keystone taxa of bacterial and fungal communities in apple pear orchard soils and their strong correlation with soil properties could provide useful clues toward sustainable management of orchards.

## 1. Introduction

The Yanbian Korean Autonomous Prefecture of Jilin Province is the largest apple pear producer in China, known as the “Hometown of Apple Pears”. Apple pears have great nutritional value and are favored by many people because they are rich in vitamin C, vitamins B_1_, B_2_, calcium, iron, phosphorus, and other nutrients and trace elements, which promote appetite and improve digestion [1]. Previous research has shown that bacterial communities in apple orchard soils were significantly correlated with the overall quality and productivity of the orchard, e.g., the size of the apple and the soluble solids concentration in apples [2]. Investigations have shown that the composition of the soil microbial community is closely related to plant productivity [3]. Similarly, it is of practical significance to investigate the soil microbial communities in apple pear orchard soils.

Soils contain many microbes (around 1 × 10^8^ cells/g for arable soils), which play an important role in soil ecological functions [4]. Microorganisms in orchard soils are conducive to the decomposition of organic matter and the release of minerals [5]. They participate in the nutrient cycle and energy flow, which may determine the overall quality of soils [6,7]. The soil microbes have symbiotic and parasitic relationships with plants and can affect their growth [8]. Soil microbial ecology may be affected by both historical factors and environmental factors. Historical factors refers to parameters such as physical barriers, species diffusion, and environmental heterogeneity over the course of history, while environmental factors refers to determinants such as pH, soil texture, organic carbon, moisture, and nutrient content. Both historical and environmental factors may be critical in shaping soil microbial communities [9,10,11,12,13]. Many studies have shown that soil pH is considered to be a key factor influencing the compositions and diversity of soil bacterial communities [14,15]. Fungi are more adaptable to changes in pH than bacteria. Fungi usually have a wider pH growth range [16], but some studies have shown that soil pH could affect soil fungal populations and fungal diversity [17,18]. In most soils, clay particles can adsorb organic carbon and nutrients and, at the same time, provide a better living environment for bacteria and fungi [19,20]. Dissolved organic carbon and dissolved nitrogen are necessary carbon and nitrogen sources for microbial growth, so they are usually significantly related to soil microbial communities [21]. Previous studies have focused more on the microbial ecology of major fruit-producing soils [22], while the study of minor fruit-producing farms is limited, e.g., the apple pear growing soils in the current study.

It is well accepted that with the increase in geographic distance, community similarities decrease [23]. The distribution of microorganisms is zonal and regional, which shows that geographic distance within a local spatial scale is an important factor in microbial communities and diversity changes [24,25]. It is reasonable to assume that there is a correlation between the impact of orchard soil properties on soil bacterial and fungal communities. Therefore, this study tried to identify the key factors affecting soil microbial communities in orchards by correlating community structure with both environmental factors and geographic distance.

Soil bacterial and fungal communities account for the vast majority of total soil biomass and they play an important role in maintaining soil functions including plant support, acting as a genetic resource bank, and pollution purification. Soil microbial communities in combination with soil physicochemical properties would enable people to evaluate the overall health, quality, and fertility of soils. Therefore, in this study, the sequencing data, soil properties, and geographic distance were combined, and the research objectives were to: (i) understand the compositions of bacterial and fungal communities, (ii) investigate keystone taxa in the network structure of bacterial and fungal co-occurrence networks, (iii) obtain the relationships between microbial communities, geographic distance, and environmental factors, and (iv) discuss potential implications for the sustainable management of orchards.

## 2. Materials and Methods

### 2.1. Soil Sample Collection and Soil Physiochemical Property Determination

A total of 28 soil samples were collected in spring (early May) from three apple pear orchards in Jilin province in northeast China (Figure S1, Table S9), 8 were from Yanji (YJ, 42°55′ N, 129°33′ E), 11 were from Longjing (LJ, 42°30′ N, 129°43′ E), and 9 were from Helong (HL, 42°42′ N, 129°8′ E). The three orchards contribute more than 60% of the apple pear output of the region. The same apple pear trees were planted in these orchards and they were subjected to similar agricultural management strategies (the apple pear orchards mainly (80%) use organic fertilizer (e.g., cattle manure) and use chemical fertilizer as a supplement (20%)). The apple pear species is *Pyrus bretschneideri* Rehd. In all apple pear orchards, the distances between plants and rows were 4 m and 5 m, respectively, i.e., 500 plants/acre. Soil samples were collected with a sterile stainless-steel shovel. Each sample was composed of 3 separate soil cores at a distance of 5 m. All soil samples were transported on ice to the laboratory within the same day and sieved to 2 mm to remove stones and plant residues. Two subsamples were then made, one subsample was air dried for soil physical and chemical properties determination as well as annual mean temperature and precipitation (Table S1), and the other subsample was stored in the refrigerator at −80 °C until DNA extraction. Soil pH was measured with a glass electrode at a soil–water ratio of 1:2.5. Electrical conductivity (EC, soil-to-water ratio, 1:2.5) was measured by a conductivity meter. Soil water soluble organic carbon (WSOC, mg/kg) was determined by UV absorbance at 254 nm [26]. Soil particle size distribution (clay, silt, and sand content in g/100 g) was measured with a laser particle size analyzer (Bettersize 2000, Dandong, China), soil total soluble nitrogen (TN, mg/kg) was determined by potassium persulfate oxidation spectrophotometry (MapData, Shanghai, China), total dissolved phosphorus (TDP, mg/kg) was determined by antimony molybdenum colorimetry (MapData, Shanghai, China), ammonium nitrogen (NH_4_^+^-N, mg/kg) was determined by Nessler’s reagent colorimetry, and nitrate nitrogen (NO_3_^−^-N, mg/kg) was measured by dual-wavelength UV spectrophotometry.

### 2.2. Soil DNA Extraction, Sequencing, and Data Analysis

Total DNA was extracted from the soil using a DNA isolation kit (Omega, Norcross, GA, USA), and DNA extraction quality was determined by 0.8% agarose gel electrophoresis. The quality and concentration of soil DNA were evaluated using a NanoDrop NC2000 spectrophotometer (Thermo Scientific, Waltham, MA, USA). According to the bacterial 16S rRNA amplicon library construction, the primers 338F: ACTCCTACGGGAGGCAGCA and 806R: GGACTACHVGGGTWTCTAAT were used to amplify the V3–V4 region of the 16S rRNA gene. In terms of fungi, the ITS1 gene was amplified using the primers ITS5F: GGAAGTAAAAGTCGTAACAAGG and ITS1R: GCTGCGTTCTTCATCGATGC [27]. The PCR amplification products were detected on 2% agarose gel electrophoresis, and the target fragments were cut and recovered. With reference to the preliminary quantitative results of electrophoresis, the PCR amplification products were quantified by fluorescence. High-throughput sequencing was performed on the Illumina Miseq platform (Illumina, San Diego, CA, USA) [28] at Shanghai Personal Biotechnology Co., Ltd. (Shanghai, China). The QIIME software (Quantitative Insights Into Microbial Ecology, v1.8.0, http://qiime.org/, accessed on 3 December 2017) [29] was used to identify interrogative sequences. Then, the QIIME software was used to call USEARCH (v5.2.236) in order to check and remove chimera sequences. The QIIME software was then applied using the UCLUST (v1.0) sequence comparison tool [30], which could merge and divide OTU sequences previously obtained according to 97% sequence similarity based on the Greengenes database (Release 13.8) for bacteria and the UNITE database (release 115) for fungi. The sequencing data have been deposited with links to BioProject under accession numbers PRJNA640242 for the bacterial community and PRJNA640241 for the fungal community in the NCBI BioProject database.

### 2.3. Statistical Methods

Graphics were constructed with OriginPro9.0 (OriginLab, Northampton, MA, USA), histograms and boxplots described the compositions and differences in bacterial and fungal communities. The differences in the relative abundance of bacterial and fungal communities among the three sampling sites were analyzed by analysis of variance (ANOVA). Pearson correlation was calculated using SPSS 22 (IBM, Armonk, NY, USA). Principal coordinates analysis (PCoA) and dissimilarity analysis were used to analyze the differences in community compositions at the three sampling points. Principal coordinates of neighbor matrices (PCNM) was used to convert latitude and longitude coordinates into geographic distance. A partial Mantel test and canonical correspondence analysis (CCA) were used to study the relationship between community structure and soil physiochemical parameters. Variation partition analysis (VPA) was used to quantify the contribution of physical and chemical properties which explain the composition of bacterial and fungal communities. PCoA, CCA, VPA, Mantel and partial Mantel test, and PCNM statistical analyses were calculated using the vegan package in R 3.5.2 [31].

### 2.4. Network Construction and Statistical Analysis

Combined with high-throughput sequencing data, molecular ecological networks (MENs) were constructed based on random matrix theory (RMT) methods, providing a way to understand network interactions in microbial communities and their responses to environmental changes [32,33]. MENs of bacteria and fungi were constructed using a network method based on random matrix theory (RMT), while network construction and acquisition of network property parameters were completed on the Molecular Ecological Network Analyses Pipeline (MENA, http://ieg4.rccc.ou.edu/mena, accessed on 15 March 2018) website [32,34,35]. MENA can be divided into two stages [32]: the first stage is the network construction, including uploading high-throughput sequencing data, standardizing relative abundance, calculating the Pearson correlation of any two OTUs, and converting the correlation matrix into similarities based on the pairwise correlation coefficient matrix, which uses a specific threshold based on random matrix theory to convert the similarity matrix into an adjacency matrix [36]. The second stage is network analysis. The network property parameters are obtained through analysis and calculation. Network property parameters include similarity threshold (st), network size (the number of OTUs), *R*^2^ of the power law, link, average connectivity (avgK), average path (GD), average clustering coefficient (avgCC), and modularity. The module has a high degree of internal connectivity and relatively few external connections. The role of each node is determined according to two attributes: within-module connectivity (*Zi*), which is used to quantify the degree of connection between a node and other nodes in the module; among module connectivity (*Pi*), which is to quantify the degree of node connection to different modules [37,38]. The role of OTU in the network is characterized by *Zi* and *Pi*, which divide all categories into four subcategories: peripherals, connectors, module hubs, and network hubs [39]. Generally, all nodes with *Zi* ≥ 2.5 or *Pi* ≥ 0.62 were designated as keystone taxa [32].

## 3. Results

### 3.1. Bacterial and Fungal Community Composition

**Figure 1:**
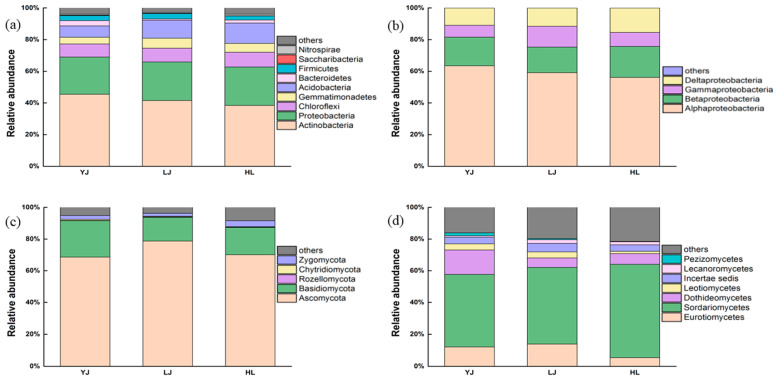
Stacked diagrams of bacterial and fungal community compositions: (**a**) bacterial phyla; (**b**) the main classes of *Proteobacteria*; (**c**) fungal phyla; (**d**) the main classes of *Ascomycota*. YJ, LJ, and HL indicate soil samples from Yanji, Longjing, and Helong, respectively.

The relative abundance of bacteria and fungi at the phylum and class levels are shown in Figure 1. The relative abundance of bacteria and fungi at the genus levels are shown in Figures S2 and S3. The number of common and unique species from Yanji, Longjing, and Helong are shown in Figure S4. The main phyla of the bacterial communities (with a relative abundance greater than 1%) in all samples were *Actinobacteria* (41.81%), *Proteobacteria* (24.07%), *Chloroflexi* (8.74%), *Gemmatimonadetes* (5.40%), *Acidobacteria* (10.44%), *Bacteroidetes* (2.03%), *Firmicutes* (3.02%), *Saccharibacteria* (0.21%), and *Nitrospirae* (0.75%), and these dominant phyla accounted for 90% of the bacterial compositions. *Proteobacteria* were mainly composed of *Alphaproteobacteria* (14.08%), *Betaproteobacteria* (4.33%), *Gammaproteobacteria* (2.66%), and *Deltaproteobacteria* (2.99%) (Figure 1b). At the genus level, bacterial communities were dominated by *Streptomyces* (3.36%), followed by *Pseudonocardia* (2.42%), *Sphingomonas* (2.21%), *Gemmatimonas* (2.16%), *Nocardioides* (1.76%), and *RB41* (1.73%).

The main phyla of fungal communities (with a relative abundance greater than 0.1%) were *Ascomycota* (72.49%), *Basidiomycota* (18.43%), *Rozellomycota* (0.20%), *Chytridiomycota* (0.43%), and *Zygomycota* (2.69%), and more than 80% of the fungal composition belonged to these phyla (Figure 1c). The relative abundance of *Ascomycota* in the three orchards was YJ (68.6%), LJ (78.8%), and HL (70.1%), respectively. Figure 1d shows the relative abundance of the main class *Ascomycota*, including *Eurotiomycetes* (10.43%), *Sordariomycetes* (50.89%), *Dothideomycetes* (9.34%), *Leotiomycetes* (3.06%), *Incertaesedis* (4.61%), *Lecanoromycetes* (1.69%), and *Pezizomycetes* (0.88%). At the genus level, fungal communities were dominated by *Penicillium* (4.86%), followed by *Humicola* (4.46%), *Guehomyces* (3.60%), *Cryptococcus* (3.18%), *Chaetomium* (2.58%), *Mortierella* (2.32%), and *Knufia* (1.82%).

**Figure 2:**
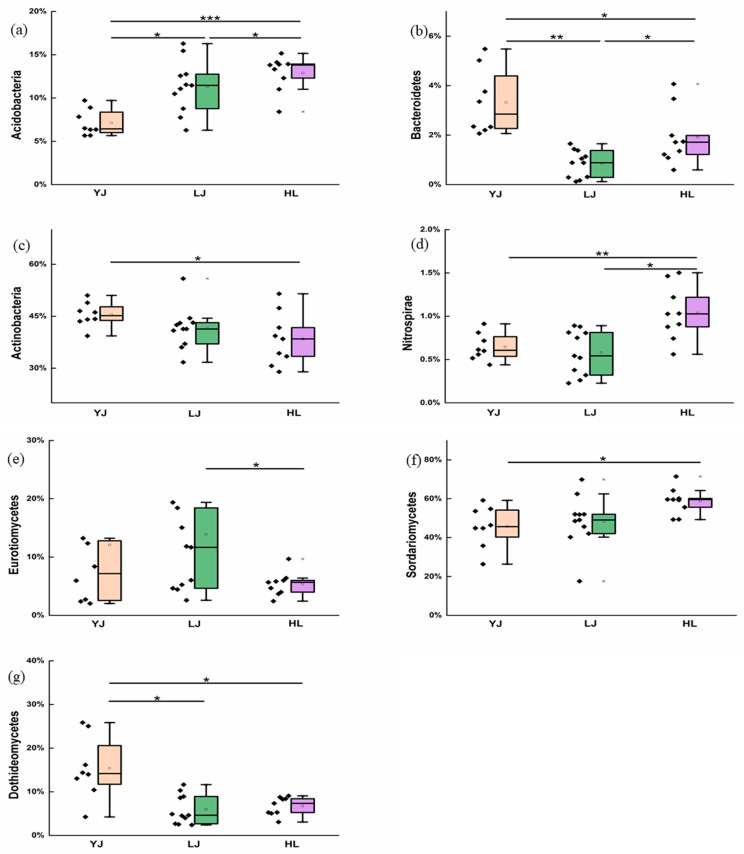
Difference analysis of percentage of relative abundance of bacterial and fungal communities in three orchard soils. (**a**–**d**) are the bacterial phyla, and (**e**–**g**) are the classes of *Ascomycota* fungi. YJ, LJ, and HL indicate soil samples from Yanji, Longjing, and Helong, respectively. *, **, and *** indicate *p* values significant at 0.05, 0.01, and 0.001, respectively.

The differences that existed in bacterial community composition among the three orchards are presented in Figure 2. The relative abundance of both *Acidobacteria* and *Bacteroidetes* demonstrated significant differences between any two orchards (*p* < 0.05, Figure 2a,b). The relative abundance of *Actinobacteria* was found to be significantly different only between HL and YJ (*p* < 0.05, Figure 2c), and the relative abundance of *Nitrospira* in HL was significantly higher than that in LJ and HL (*p* < 0.05, Figure 2d).

Regarding the fungal community, no difference was found for the relative abundance of the phylum *Ascomycota* in all three orchards, thus further analysis at class level of the phylum *Ascomycota* was performed. It was found that the relative abundance of *Eurotiomycetes* in LJ is higher than that in HL (*p* < 0.05, Figure 2e), and the relative abundance of *Sordariomycetes* in YJ is lower than that in HL (*p* < 0.05, Figure 2f). In YJ soils, the relative abundance of *Dothideomycetes* was greater than those in HL and LJ (*p* < 0.05, Figure 2g).

**Figure 3:**
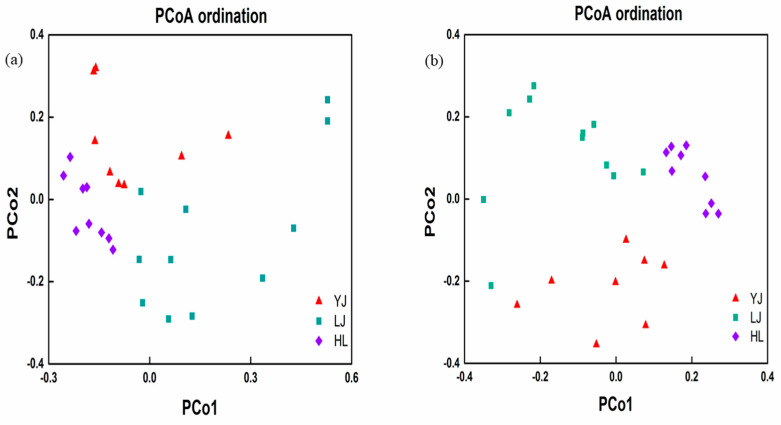
Principal coordinates analysis (PCoA) of bacterial (**a**) and fungal (**b**) communities in orchards. Red triangles, green squares and purple diamonds represent soil samples from Yanji (YJ), Longjing (LJ) and Helong (HL), respectively.

The bacterial and fungal community compositions of all soil samples were visualized via principal coordinates analysis (PCoA). Figure 3a,b revealed that the three sites YJ, LJ, and HL could be well separated by PCo1 and PCo2. A combination of different dissimilarity analysis methods including mrpp, adonis, and anosim of the three sites using Bray–Curtis distance indicated that the structure and composition of bacterial and fungal communities were significantly different (*p* < 0.01, Table 1).

### 3.2. Network Analysis

**Figure 4:**
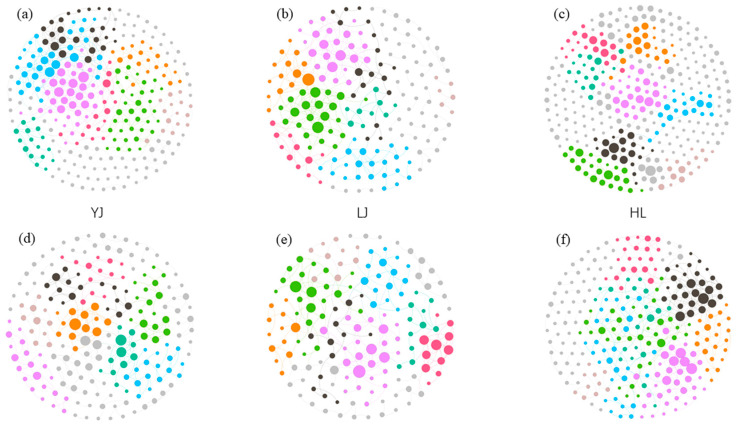
Molecular ecological network diagram visualizing interactions between bacterial communities (**a**–**c**) and fungal communities (**d**–**f**). YJ, LJ, and HL indicate soil samples from Yanji, Longjing, and Helong, respectively. The larger circles indicate that the OTU had more links with other OTUs. The circles sharing the same color indicate that those circles belonged to the same module.

Specifically, in the analysis of bacterial community, only the operational taxonomic units (OTUs) appearing in >90% of the total samples were used for network calculations. The overall topological indices (Table 2) showed that all the curves of the network connectivity distribution completely fit with the power law model (*R*^2^ values from 0.80 to 0.93). All the modularity values ranged from 0.70 to 0.88, the GD values ranged from 5.65 to 9.86, and the avgCC values ranged from 0.19 to 0.28, which were significantly higher than the three values of the randomized network, respectively. From Table 2, with the combination of the molecular ecological network (Figure 4a–c), it can be seen that LJ had the fewest nodes and edges among the three orchards, YJ’s nodes were fewer in number than HL’s, but YJ’s edges were more numerous than HL’s, and HL’s modularity was the largest of the three.

In the analysis of the fungal community, only the operable taxonomic units (OTUs) appearing in >60% of the total samples were involved in the network calculation (Figure 4d–f), and all the curves of the network connectivity distribution were fully consistent with the power law model (*R*^2^ values from 0.84 to 0.89, Table 2). All modular values ranged from 0.74 to 0.86, the GD values ranged from 5.62 to 6.73, and the avgCC values ranged between 0.13 and 0.24, which were significantly higher than those three values of the random network. LJ had the fewest nodes and edges in the three orchards. The numbers of nodes of HL and YJ are similar, but the edges of HL were more numerous than those of YJ. YJ’s network was the most modular of the three network diagrams.

**Figure 5:**
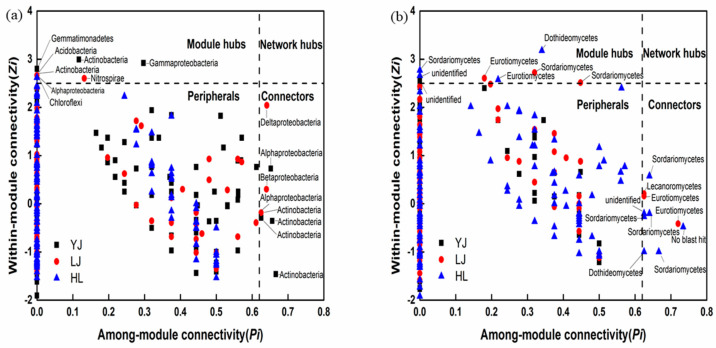
*Zi*–*Pi* diagram showing the topological distribution of OTUs based on bacterial (**a**) and fungal networks (**b**). The classification thresholds of *Zi* and *Pi* of the OTUs are 2.5 and 0.62, respectively. YJ, LJ, and HL indicate soil samples from Yanji, Longjing, and Helong, respectively.

According to within-module connectivity (*Zi*) and among-module connectivity (*Pi*), the majority (more than 95%) of the bacteria’s OTUs were on the periphery (Figure 5a). The number of peripheral OTU links was small and they were connected to the nodes in their own module. A total of eight nodes in the three orchards were in connectors (four nodes in YJ, four nodes in LJ), and eight nodes were in module hubs (three nodes in YJ, three nodes in LJ, and two nodes in HL). The nodes in connectors and module hubs were as follows: *Actinobacteria*, *Alphaproteobacteria*, *Gammproteobacteria*, and *Gemmatimonadetes* in YJ soils; *Actinobacteria*, *Deltaproteobacteria*, *Betaproteobacteria*, *Alphaproteobacteria*, *Acidobacteria*, and *Nitrospirae* in LJ soils; and *Alphaproteobacteria* and *Chloroflexi* in HL soils.

In the fungal network diagram, most (>95%) of the OTUs were on the periphery (Figure 5b). A total of 10 nodes in the three orchards were in connectors (three nodes in LJ and seven nodes in HL), and a total of eight were in module hubs (one node in YJ, three nodes in LJ, and four nodes in HL). All nodes in connectors and module hubs were exclusively affiliated with the specific *Ascomycota* phylum. An unidentified class in Yanji; *Eurotiomycetes*, *Sordariomycetes*, and *Lecanoromycetes* in LJ; and *Sordariomycetes*, *Dothideomycetes*, *Eurotiomycetes*, and an unidentified class in HL soils.

### 3.3. Relationship of Microbial Communities to Physicochemical Properties and Geographic Distance

Mantel and partial Mantel tests were used to investigate the relationship between the environmental attributes and the microbial community structure (Table 3). Mantel tests based on Bray–Curtis and Euclidian distances show that soil texture (clay, silt, and sand content), pH, WSOC, and NO_3_^−^-N are significantly correlated with bacterial community (*p* < 0.05). Clay and pH are significantly correlated with fungal communities (*p* < 0.05). The results of the Pearson correlation (Table S2) between bacterial communities and physicochemical properties show that sand and silt contents are positively correlated with PCo1 (*p* < 0.05), pH has a negative correlation with PCo1 (*p* < 0.01), and WSOC and NO_3_^−^-N are negatively correlated with PCo2 (*p* < 0.01). According to the Pearson correlation between fungal communities and soil properties (Table S3), pH had a positive correlation with PCo1 (*p* < 0.01) and a negative correlation with PCo2 (*p* < 0.05).

**Figure 6:**
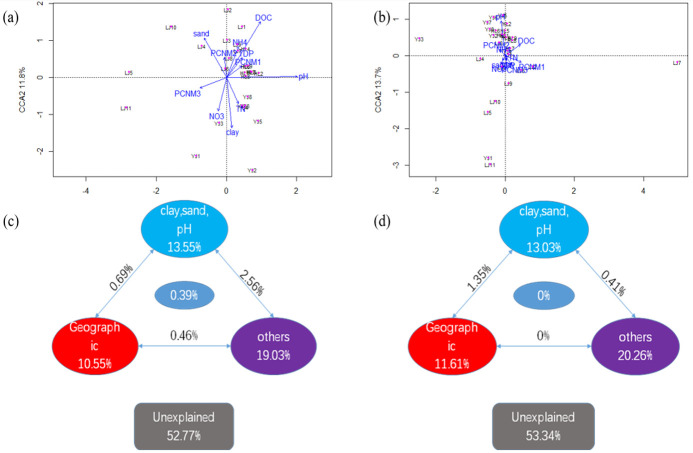
Canonical correspondence analysis (CCA) (**a**,**b**) and variation partition analysis (VPA) (**c**,**d**) explored the relationships among soil properties, geographic distance, and bacterial communities (**a**,**c**) or fungal communities (**b**,**d**).

Canonical correlation analysis (CCA) and variation partition analysis (VPA) were used to quantify the relationships among the environmental attributes, geographic distance, and the microbial community structure. The relationships among soil parameters, geographic distance, and soil bacterial community structure are shown in Figure 6a,c. These variables explained nearly 47.23% of the overall variations in the bacterial communities, with geographic distance, soil texture and pH (including clay, sand, and pH), and other physical and chemical properties (WSOC, TN, NH_4_^+^-N, NO_3_^−^-N, and TDP) explaining 10.55%, 13.35%, and 19.03%, respectively. For fungal communities (Figure 6b,d), geographic distance, soil texture and pH (clay, sand, and pH), and other soil properties could explain 11.61%, 13.03%, and 20.26%, respectively.

## 4. Discussion

### 4.1. Soil Microbial Composition and Keystone Taxa

The composition and structure of soil bacterial and fungal community in apple pear orchards were characterized by high-throughput sequencing and the keystone taxa were identified by molecular ecological network analysis based on within-module connectivity and among-module connectivity. Among the bacterial communities, *Actinobacteria* and *Proteobacteria* accounted for the highest proportion. *Alphaproteobacteria* and *Betaproteobacteria* accounted for the highest proportion of *Proteobacteria*, which was consistent with a previous study on bacterial community composition of apple orchards [2]. Manuel’s research on major global bacterial systems found that *Proteobacteria* and *Actinobacteria* were the two bacterial phyla with the highest relative abundances [3], which was consistent with the results of our study showing that *Actinobacteria* and *Proteobacteria* were also the keystone taxa in the bacterial communities of the apple pear orchards. Therefore, *Actinobacteria* and *Proteobacteria* were both dominant phyla and keystone taxa, indicating that they might play an essential role in the ecological functioning of microbial communities. Similar findings were also found for rhizospheric and bulk soils, subtropical, and low temperate soils [21,40]. In addition to *Actinobacteria* and *Proteobacteria*, the keystone taxa of the three orchards included *Acidobacteria*, *Nitrospirae*, and *Chloroflexi*. *Acidobacteria* in the soil was usually considered to be negatively correlated with pH, so this may be the reason why *Acidobacteria* became keystone taxa in LJ soils with relatively lower pH. *Nitrospirae* were keystone taxa in the bacterial communities, and they could convert ammonia nitrogen into nitrate nitrogen. This conversion directly affected the ratio of nitrate nitrogen to ammonia nitrogen in the soil, thus playing an important role in nitrogen cycling in apple pear orchard soils. *Chloroflexi* are bacteria that produced energy through photosynthesis and have facultative anaerobic characteristics [41]. Light intensity might be the main reason why *Chloroflexi* became keystone taxa of the bacterial community. For the fungal community, *Ascomycota* and *Basidiomycota* were the dominant phyla, and all keystone taxa identified belonged to *Ascomycota* according to the *Zi-Pi* diagram. Studies have shown that *Ascomycota* are dominant in soil fungal communities [42]. *Ascomycota* are mostly saprophytes and play an important role in degrading soil organic matter. *Basidiomycota* are saprophytic or parasitic. In soils with higher moisture, they can decompose lignocellulose. Previous research has shown that *Ascomycota* and *Basidiomycota* are the main decomposers in soils [43,44]. Alternatively, at the genus level, some fungi such as *Phoma* and *Penicillium* were found to have pathogenic characteristics that may be associated with fruit spoilage [45,46]. *Sphingomonas*, *Bacillus*, and *Streptomyces* are the dominant genera in soils, which is similar to other studies [47,48,49].

The keystone taxa generally played an important role in structuring and functioning of the microbial community and such a role might not change due to their abundance [50]. Moreover, the positions of the keystone taxa in the microbial community are very special, and their extinction would bring huge changes to the structures and functions of the microbial communities [50]. The importance of keystone taxa might be related to the complexity of biological processes, e.g., nitrogen fixation or organic carbon turnover. When the processes are carried out by some special microbes, the impact of keystone taxa might be more direct and obvious [51]. The keystone taxa might affect microbial communities in different ways: (1) they might act via indirect groups, which could selectively adjust their abundance to regulate community structure and function by producing metabolites, bacteriocins, or toxins to change community composition and structure [52,53]; (2) they might excrete antibiotics to selectively alter the composition and structure of microbial communities; (3) they might also establish synergies and change the number of their partners, which might have an major impact on microbial community structure, composition, and ecological functioning [50].

### 4.2. Effects of Soil Properties on Microbial Communities

The results of the Mantel and partial Mantel tests show that soil texture, pH, WSOC, and NO_3_^−^-N can significantly affect the composition of bacterial communities, while soil texture and pH can significantly influence the composition of fungal communities. Obviously, soil texture is an important factor in shaping both bacterial and fungal communities in apple pear orchards. Clay could serve as a binding site for microorganisms and provide nutrients, adjust the soil pH range, absorb harmful metabolites in the environment, as well as protect microorganisms from external adversity and predators [54]. Soil texture has a stronger impact on bacterial communities than on fungal communities (Table 3). This is because bacteria mainly exist in soil particles, while fungi mainly live in soil aggregates and most could not survive in micropores as they are larger than bacteria [55,56]. This study found that sand showed a significantly positive relation to PCoA1, which could be explained by the spatial isolation hypothesis [57]. This hypothesis proposes that when the sand content is relatively large, the soil pores appear to be larger, and water forms a water film in the pores, so many hydrated microhabitats appear in the soil, which helps to increase the bacterial diversity [57]. While most fungi live in soil aggregates, so the soil texture has less impact on fungi than on bacteria. pH is also an important factor affecting soil bacterial and fungal communities, which is consistent with previous research results [58]. When the soil pH is too high or too low, it is a direct stress to the soil microbial communities as an external adversity, and pH can also directly determine the speciation and bioavailability of the mineral nutrients. The current research also shows that soil pH has a significant effect on the bacteria *Deltaproteobacteria* and *Saccharibacteria* of the fungi *Rozellomycota*. Previous studies have found that the relationship between fungi and pH was weaker than that between bacteria and pH [59], which is roughly consistent with our results. The composition of the bacterial community is also affected by WSOC and NO_3_^−^-N. According to the results of Mantel and partial Mantel tests, the impact of WSOC on the bacterial community could be explained from two perspectives: one is that WSOC directly serves as the carbon source for bacteria, and the other is that WSOC indirectly influences bacterial communities by changing soil properties, e.g., the average size of soil aggregates [60,61]. It was also found that NO_3_^−^-N affects the bacterial community by affecting other soil properties, and might not directly affect the bacterial community. These results were somewhat in contrast to those from other studies [62,63], the potential reason might lie in the amount and history of nitrate-containing fertilizer applied to apple pear orchard soils [64].

### 4.3. Interactions between Bacterial Taxa as Revealed by Co-Occurrence Networks

MENs are widely used in ecological studies to compare networks under different yields and different management modes, to compare networks of bacteria, fungi, and archaea, as well as to identify symbiotic and trophic interactions between species within a network [65,66,67,68]. An ecological network reflects complex biological interactions in the ecosystem, with nodes representing species and edges representing interactions between species [62,69]. Since nodes in a network represent species in the community, the topological role of different nodes could be described as the basis for determining keystone taxa. The keystone taxa are highly related taxa, and they have a considerable impact on the structure and function of the microbiome; therefore, if they were to be removed, rapid changes would occur in the structure and function of the microbiome [50]. Our data provided some useful insights into a better understanding of the soil microbial community structure of apple pear orchards and the keystone taxa in the bacterial and fungal communities. Such information would be helpful in designing managerial strategies for apple pear orchard soils.

### 4.4. Effects of Geographic Distance on Microbial Communities

It can be seen from CCA and VPA that geographic distance could significantly affect bacterial and fungal communities, as it can explain 10.55% of bacterial community differences and 11.61% of fungal community differences. Environmental selection and geographic distance had usually been considered as the two main ecological processes affecting the overall variation in microbial communities [11]. The current study showed that some of the variation in orchard soil bacterial and fungal communities are due to geographic distance, which is consistent with previous research results [62]. It is a common phenomenon that the similarity of community structure decreases with the increase in geographic distance. The main driving force of this biogeographic pattern is diffusion limitation [23].

The geographic distance of the three orchard samples was relatively close, but the microbial community differences were significant (Table 1). This might be due to the major differences in regional microclimate, especially the mean annual precipitation, which is much higher in HL soils (Table S1). In this study, although soil characteristics (such as soil texture, pH, WSOC) and geographic distance can explain about 50% of overall variation in bacterial and fungal communities, leaving another 50% unexplained, which might be attributed to the unmeasured soil parameters, including soil moisture, redox potential, soil virus, protozoa, as well as soil animal communities. Obviously, additional work is needed to gain a better understanding of the factors shaping apple pear orchard soil microbial communities.

In the current work, soil physical, chemical, and biological factors, as well as geographic distance were found to be important parameters in shaping the composition and structure of both bacterial and fungal communities in apple pear orchard soils. However, all of these factors only explained less than 70% of the overall variation in microbial communities across all samples. The possible reasons might lie in the unmeasured soil properties, including but not limited to soil minerals, trace metals, available P and K, organic N, soil animals, protists, and viruses. The local climate should also be taken into account. Previous studies revealed that local anthropogenic activities and climate change might exert a major influence on soil quality as indicated by soil enzymatic activities [22,70].

## 5. Conclusions

In the current study, high-throughput sequencing in combination with bioinformatics analysis techniques were applied to probe the composition and structure of bacterial and fungal communities in apple pear orchard soils. The keystone taxa were identified by the topological properties of molecular ecological networks. We also found that soil properties and geographic distance significantly affected bacterial and fungal community compositions. Soil texture and pH significantly affected the compositions of bacterial and fungal communities, and bacterial communities were also affected by WSOC and NO_3_^−^-N. The results of our study provided useful background information for apple pear orchard soil management in order to improve the overall productivity and quality of apple-pear.

## Figures and Tables

**Table 1:** Dissimilarity analysis based on Bray–Curtis distance of bacteria and fungi in three orchards. Three distance indices are mrpp (*δ*), adonis (*F*), and anosim (*R*). MRPP, multi response permutation procedure; Anosim, analysis of similarities; adonis, permutational multivariate analysis of variance.

	Mrpp		Adonis		Anosim	
	*δ*	*p*	*F*	*p*	*R*	*p*
bacteria	0.684	0.001	2.193	0.002	0.407	0.001
fungi	0.656	0.001	2.789	0.001	0.369	0.001

**Table 2:** Topological properties of empirical molecular ecological networks (MENs) of bacterial and fungal communities and their related random MENs.

		Bacterial Community			Fungal Community		
		YJ	LJ	HL	YJ	LJ	HL
Empirical networks	st	0.88	0.86	0.87	0.9	0.85	0.9
	Network size	275	177	324	201	157	253
	link	491	287	383	252	216	403
	avgK	3.57	3.24	2.36	2.51	2.75	3.19
	GD	7.33	5.65	9.86	6.73	6.23	5.62
	avgCC	0.28	0.2	0.19	0.24	0.13	0.14
	Modularity	0.71	0.7	0.88	0.86	0.78	0.74
	*R* ^2^	0.86	0.93	0.8	0.87	0.84	0.89
Random networks	GD ± SD	4.23 ± 0.06	4.07 ± 0.08	6.60 ± 0.18	5.40 ± 0.16	4.80 ± 0.14	4.45 ± 0.08
	avgCC ± SD	0.019 ± 0.006	0.023 ± 0.008	0.005 ± 0.003	0.009 ± 0.006	0.014 ± 0.008	0.016 ± 0.006
	Modularity ± SD	0.54 ± 0.01	0.55 ± 0.01	0.74 ± 0.01	0.69 ± 0.01	0.63 ± 0.01	0.58 ± 0.01

st, similarity threshold; network size, the nodes in a network; avgK, average connectivity; GD, average path; avgCC, average clustering coefficient. YJ, LJ, and HL indicate soil samples from Yanji, Longjing, and Helong, respectively.

**Table 3:** Mantel and partial Mantel tests assessed the importance of environmental factors in the entire network of bacterial and fungal communities.

	Bacterial Community		Fungal Community	
	Mantel	Partial	Mantel	Partial
clay	0.232 **	0.117	0.172 *	0.152
silt	0.352 ***	0.284 *	0.158	0.140
sand	0.335 **	0.236 *	0.177	0.159
pH	0.555 ***	0.573 ***	0.277 *	0.277 *
WSOC	0.236 **	0.174 *	0.082	0.063
NO_3_^−^-N	0.263 *	0.042	0.054	−0.020

WSOC, water soluble organic carbon; NO_3_^−^-N, nitrate nitrogen. *, **, and *** indicate *p* values are significant at 0.05, 0.01, and 0.001, respectively.

## Data Availability

The sequencing data have been deposited with links to BioProject under accession number PRJNA640242 for the bacterial community and PRJNA640241 for the fungal community in the NCBI BioProject database.

## Supplementary Materials

The following supporting information can be downloaded at: https://www.mdpi.com/article/10.3390/microorganisms12091751/s1, Figure S1: Map of sampling sites; Figure S2. The relative abundance of bacteria at genus level from Yanji (YJ1–8), Longjing (LJ1–11), and Helong (HL1–9); Figure S3. The relative abundance of fungus at genus level from Yanji (YJ1–8), Longjing (LJ1–11), and Helong (HL1–9); Figure S4. Common and unique OTUs of bacteria (a) and fungi (b) from YJ (Yanji), LJ (Longjng), and HL (Helong); Table S1: Soil properties and climate conditions of sampling area; Table S2: Total bacterial sequences, OTU counts, Chao1 index, and Shannon index of each sample from apple pear orchards located in Yanji, Longjing, and Helong; Table S3: Total fungal sequences, OTU counts, Chao1 index, and Shannon index of each sample from apple pear orchards located in Yanji, Longjing, and Helong; Table S4: Number of bacterial taxa at phylum, class, order, family, genus, and species levels identified in samples from different apple pear orchards; Table S5: Number of fungal taxa at phylum, class, order, family, genus, and species levels identified in samples from different apple pear orchards; Table S6: Taxonomy corresponding to key OTUs in *Z*i*-P*i diagram; Table S7: Pearson correlation between bacterial communities and physicochemical properties; Table S8: Pearson correlation between fungi communities and physicochemical properties; Table S9. Latitude and longitude of sampling sites.
