# Supplementary material for: Variation in Bacterial and Fungal Communities in Soils from Three Major Apple Pear (Pyrus bretschneideri Rehd.) Orchards
Source: Microorganisms. 2024 Aug 23;12(9):1751. doi: 10.3390/microorganisms12091751 (PMC11434001; doi:10.3390/microorganisms12091751)
Supplement: Supplementary file 1 [file microorganisms-12-01751-s001.zip › microorganisms-3164200-supplementary.pdf]

Supporting Information

Variation of Bacterial and Fungal Communities in Soils from Three Major Apple-Pear (*Pyrus bretschneideri Rehd.*) Orchards

Guangze Lyu [1,2], Jiayang Hu[2], Jincai Ma[2*]

[1] Key Laboratory of Ground Water Resource and Environment, Ministry of Education, Jilin University, Changchun 130021, China;

[2] Jilin Provincial Key Laboratory of Water Resources and Environment, Jilin University, Changchun 130021, China

*to whom correspondences should be addressed

Jincai Ma

130012

E-mail: jincaima@jlu.edu.cn

Phone: +86-431-85168429

College of New Energy and Environment

Jilin University

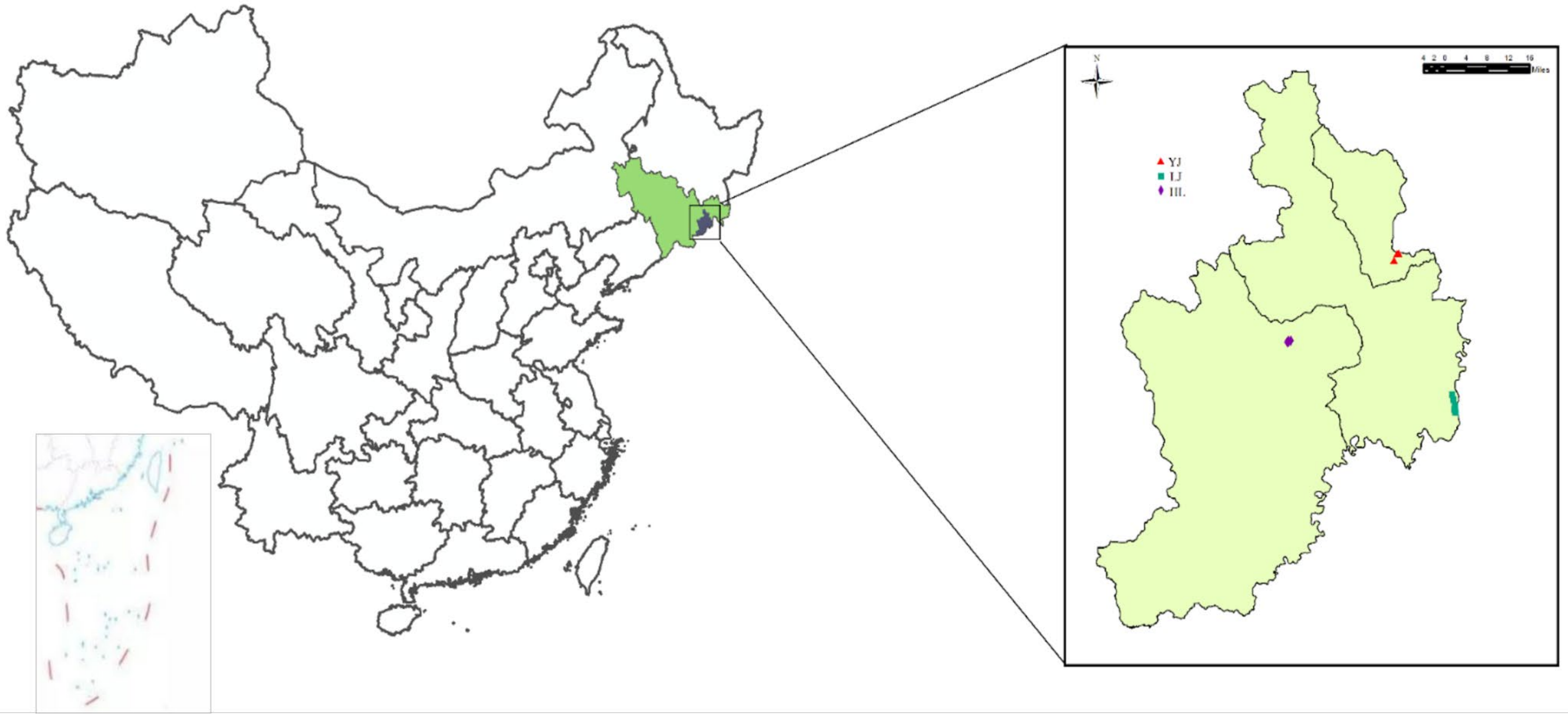

Figure S1. Map of sampling sites. YJ, LJ, and HL indicate Yanji, Longjing, and Helong, respectively**.**

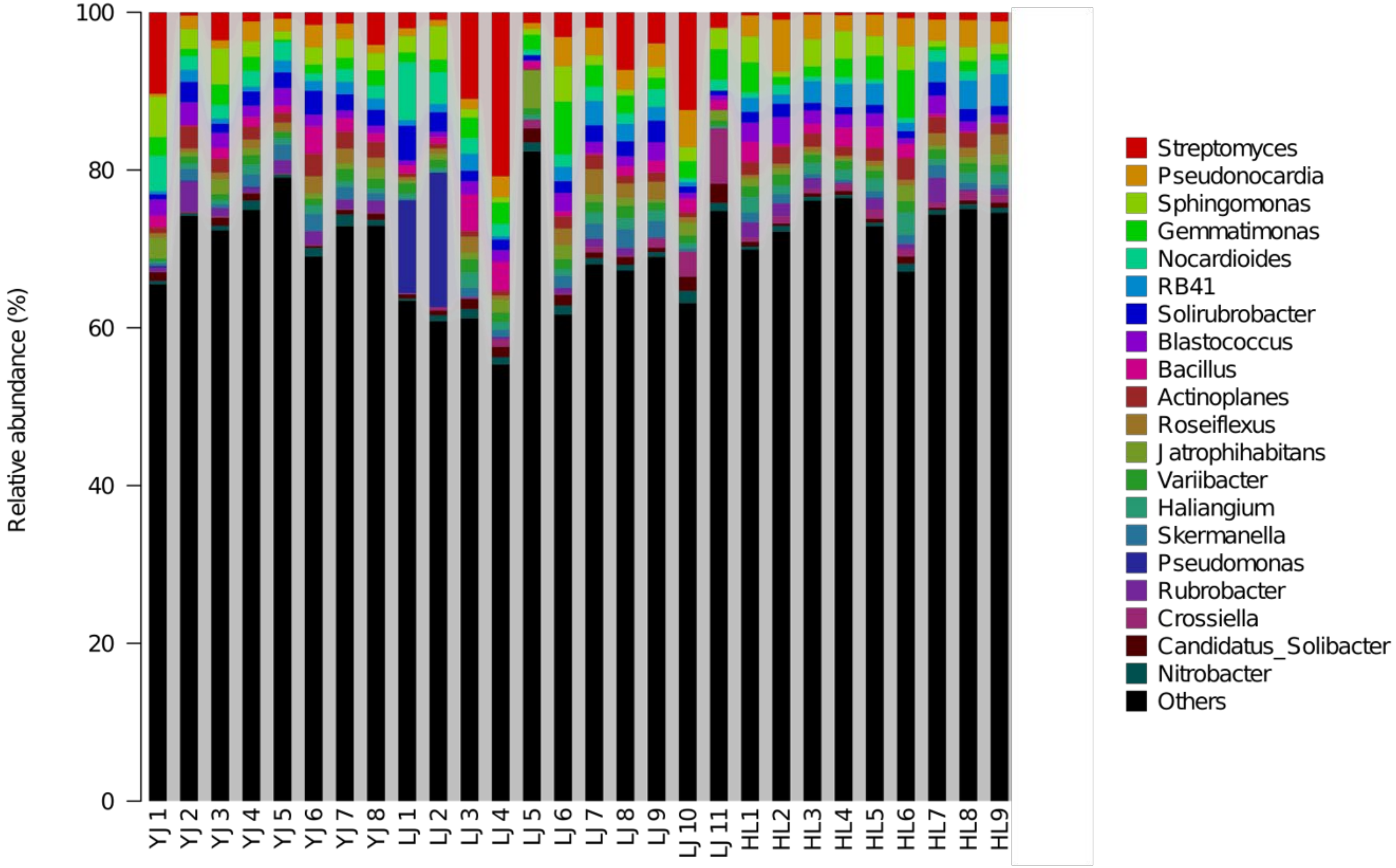

Figure S2. The relative abundance of bacteria at genus level from Yanji(YJ1-8), Longjing (LJ1-11) and Helong(HL1-9)

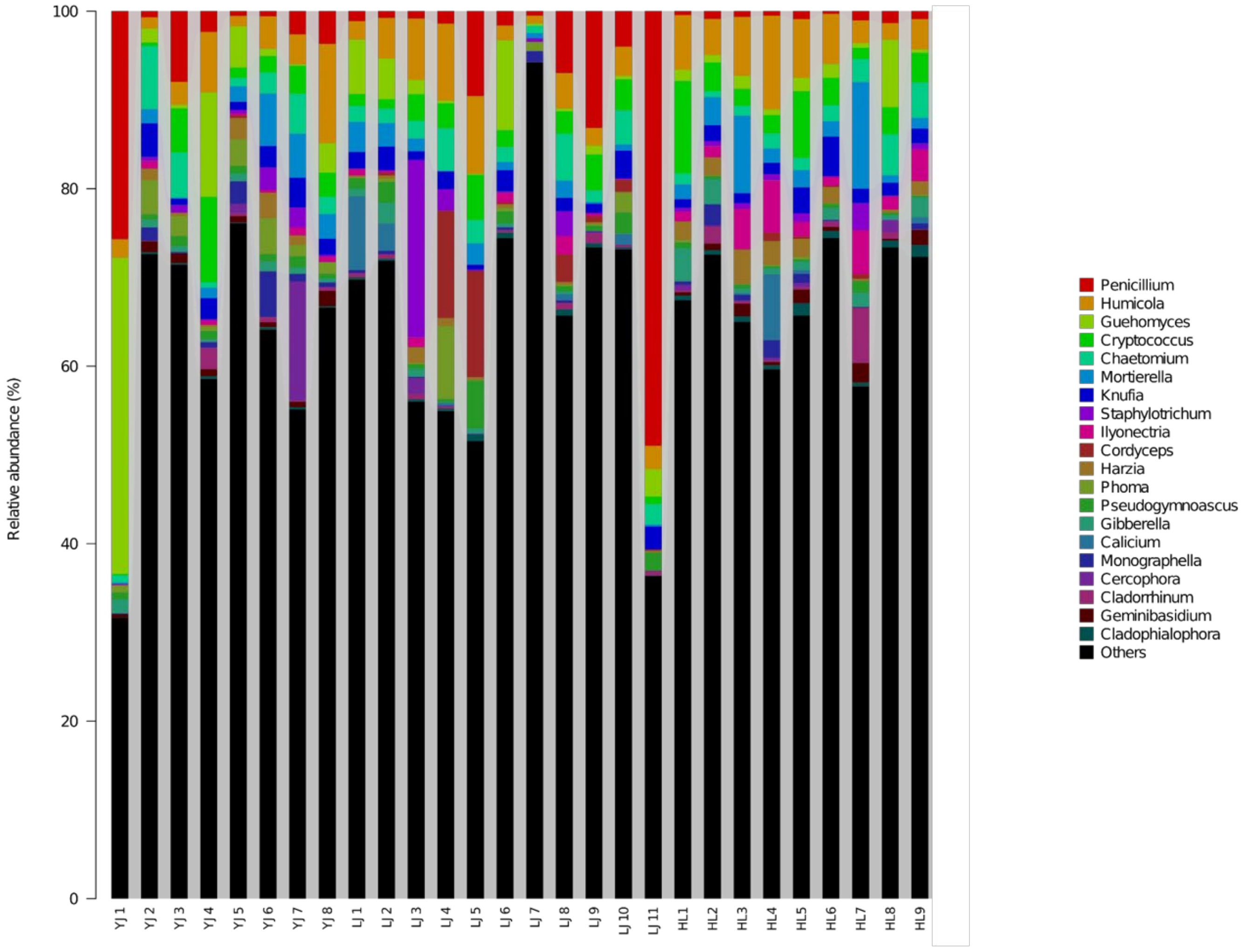

Figure S3. The relative abundance of fungus at genus level from Yanji(YJ1-8), Longjing (LJ1-11) and Helong(HL1-9)

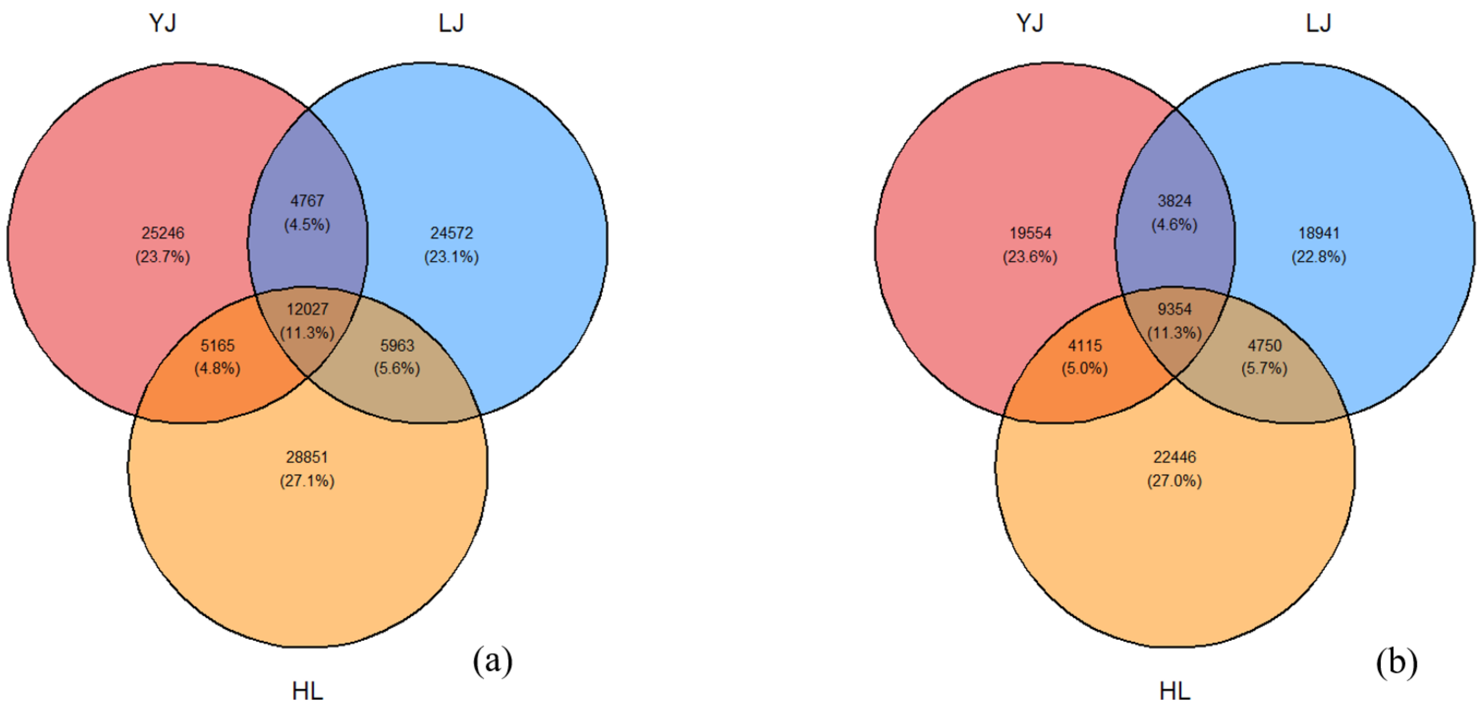

Figure S4. Common and unique OTUs of bacteria (**a**) and fungi (**b**) from YJ (Yanji), LJ (Longjng)and HL(Helong)

Table S1. Soil properties and climate conditions of sampling area. MAT, MAP represented mean annual temperature and precipitation, respectively. EC, electrical conductivity, WSOC, water soluble organic carbon, TN, soluble total nitrogen, TDP, total dissolved phosphorus, NH_4^+-N, ammonium nitrogen, NO_3^--N, nitrate nitrogen

	MAT ºC	MAP mm	Altitude m	Sample	EC mS/cm	Clay g/100g	Silt g/100g	Sand g/100g	pH	WSOC mg/kg	TN mg/kg	NH_4^+ mg/kg	NO_3^- mg/kg	TDP mg/kg
Yanji	5.8	527	227	YJ1	124.60	21.34	72.29	6.36	6.03	7.96	25.45	2.46	20.61	7.43
				YJ2	220.50	18.52	73.94	7.54	6.82	11.51	48.43	1.88	37.05	1.88
				YJ3	58.50	15.11	74.56	10.34	6.97	19.47	7.70	4.25	2.50	3.63
				YJ4	95.30	17.68	72.52	9.80	6.77	19.76	19.63	3.09	7.77	3.14
				YJ5	105.95	10.18	62.40	27.42	7.13	10.92	15.93	1.69	5.73	1.64
				YJ6	99.75	13.20	72.20	14.60	7.10	17.33	17.34	1.98	10.28	2.24
				YJ7	96.10	14.22	74.76	11.02	7.04	15.20	13.17	1.74	6.26	1.82
				YJ8	65.00	16.89	72.88	10.24	7.05	21.94	7.76	1.57	0.83	1.28
Longjing	5.6	549	244	LJ1	305.00	8.96	65.71	25.33	6.89	39.42	52.67	5.56	40.68	7.07
				LJ2	69.20	10.38	65.47	24.15	6.71	35.73	10.11	2.73	3.83	6.16
				LJ3	42.60	13.48	74.07	12.45	6.77	35.73	8.70	3.12	0.50	1.94
				LJ4	34.55	10.36	63.03	26.60	6.83	23.35	7.58	3.55	0.50	2.90
				LJ5	82.50	12.44	63.61	23.95	5.98	10.34	17.63	2.32	13.48	2.42
				LJ6	65.35	16.84	75.08	8.08	6.33	36.80	14.93	4.64	3.43	2.54
				LJ7	56.15	16.30	74.34	9.36	6.46	37.29	15.87	2.97	1.35	2.72
				LJ8	57.85	15.45	76.08	8.47	6.44	33.74	12.52	4.42	3.54	2.18
				LJ9	52.05	10.26	67.28	22.46	6.81	30.97	7.52	3.58	0.50	8.21
				LJ10	26.65	9.91	57.50	32.60	5.98	19.23	8.35	2.97	0.99	2.54
				LJ11	84.65	17.70	74.23	8.07	5.73	10.54	14.11	2.51	12.16	2.78
Helong	4.8	734	370	HL1	59.45	18.88	73.82	7.30	6.88	40.20	4.82	3.99	0.50	4.05
				HL2	91.25	15.32	75.02	9.66	7.11	47.00	18.99	7.13	2.07	7.43
				HL3	58.80	13.25	74.04	12.71	7.06	26.90	8.52	3.79	1.46	7.55
				HL4	92.23	13.80	74.66	11.54	7.08	29.52	13.70	3.31	2.46	8.87
				HL5	66.20	18.13	74.29	7.58	7.06	27.72	13.70	3.53	0.91	3.66
				HL6	64.50	16.49	74.90	8.61	6.78	24.52	13.40	3.29	1.23	2.12
				HL7	76.85	14.09	71.93	13.98	6.91	19.13	20.63	3.10	4.60	8.93
				HL8	86.35	19.91	73.34	6.75	6.77	19.08	22.45	2.51	7.70	2.54
				HL9	69.93	14.91	74.57	10.52	6.82	18.64	20.63	1.84	0.92	3.81

Table S2. Total bacterial sequences, OTU counts, Chao1 index, and Shannon index of each sample from apple-pear orchard located in Yanji, Longjing, and Helong

Orchard	Sample	Sequence	OTU	*Chao*1	*Shannon*
Yanji	YJ1	36233	22284	3591	10.32
	YJ2	30996	15531	4567	11.00
	YJ3	37904	23366	4160	10.86
	YJ4	31196	15352	2125	10.59
	YJ5	32055	16933	2308	10.61
	YJ6	31631	15512	2153	10.55
	YJ7	33551	16499	2284	10.71
	YJ8	33620	17202	2431	10.73
Longjing	LJ1	36996	21339	4528	10.22
	LJ2	32011	19298	4086	9.77
	LJ3	36231	20023	4033	10.45
	LJ4	27056	16714	2219	10.07
	LJ5	33266	21181	2373	9.66
	LJ6	35648	18950	3133	10.70
	LJ7	28214	17352	2679	10.70
	LJ8	27260	16598	2445	10.60
	LJ9	30914	15845	2120	10.52
	LJ10	38786	21187	3780	10.32
	LJ11	33778	20695	2816	9.76
Helong	HL1	44101	26943	3779	10.84
	HL2	32995	16350	2294	10.71
	HL3	45611	30583	3123	10.77
	HL4	48568	26078	4229	10.97
	HL5	44611	26240	3920	10.89
	HL6	27774	14782	2188	10.61
	HL7	27590	14683	2196	10.59
	HL8	29691	16363	2376	10.75
	HL9	29407	14831	2287	10.57

Table S3. Total fungal sequences, OTU counts, Chao1 index, and Shannon index of each sample from apple-pear orchard located in Yanji, Longjing, and Helong

Orchard	Sample	Sequence	OTU	*Chao*1	*Shannon*
Yanji	YJ1	43975	43084	395	4.85
	YJ2	39591	38745	540	5.78
	YJ3	44081	42243	475	5.68
	YJ4	45121	44350	571	6.19
	YJ5	44554	43269	552	6.73
	YJ6	45717	44598	681	6.62
	YJ7	46514	45297	586	6.40
	YJ8	44649	43511	505	6.36
Longjing	LJ1	45137	44250	639	7.02
	LJ2	43529	42305	426	6.99
	LJ3	43701	41230	503	6.16
	LJ4	44732	43382	535	5.49
	LJ5	45153	43721	355	5.74
	LJ6	44997	43982	564	6.31
	LJ7	41139	38131	212	6.73
	LJ8	47718	46006	558	6.26
	LJ9	45405	42684	593	6.31
	LJ10	47050	44519	466	5.84
	LJ11	47622	46371	318	4.96
Helong	HL1	45655	42195	631	5.96
	HL2	46059	44176	553	6.07
	HL3	50355	44164	607	6.85
	HL4	50355	44212	614	6.12
	HL5	37234	36659	593	6.28
	HL6	38412	35732	408	6.44
	HL7	45669	43601	524	6.27
	HL8	42735	40900	523	6.87
	HL9	39621	37751	464	5.99

Table S4 Number of bacterial taxa at Phylum, Class, Order, Family, Genus, and Species levels identified in samples from different apple-pear orchards

Orchard	Sample	Phylum	Class	Order	Family	Genus	Species
Yanji	YJ1	17	52	78	158	284	156
	YJ2	24	68	88	170	282	135
	YJ3	21	65	99	190	318	170
	YJ4	18	58	79	151	241	112
	YJ5	20	70	96	174	306	113
	YJ6	16	49	70	140	228	109
	YJ7	15	52	68	134	223	105
	YJ8	18	58	84	157	249	132
Longjing	LJ1	17	62	85	173	272	139
	LJ2	20	72	103	184	294	138
	LJ3	17	60	87	155	242	122
	LJ4	16	49	83	155	219	115
	LJ5	22	67	96	164	189	98
	LJ6	17	56	83	149	228	112
	LJ7	20	61	80	152	221	106
	LJ8	16	57	85	151	216	103
	LJ9	17	54	79	157	230	102
	LJ10	19	62	90	159	232	129
	LJ11	17	57	97	172	255	130
Helong	HL1	20	63	91	176	269	129
	HL2	15	56	78	144	195	99
	HL3	22	72	99	173	241	111
	HL4	21	69	99	183	276	143
	HL5	22	69	99	177	254	132
	HL6	16	63	79	147	187	99
	HL7	18	61	85	154	233	112
	HL8	23	72	97	171	248	121
	HL9	22	74	94	162	215	112

Table S5. Number of fungal taxa at Phylum, Class, Order, Family, Genus, and Species levels identified in samples from different apple-pear orchards

Orchard	Sample	Phylum	Class	Order	Family	Genus	Species
Yanji	YJ1	8	17	41	55	74	151
	YJ2	9	22	50	72	117	217
	YJ3	8	18	45	56	91	173
	YJ4	8	19	57	84	140	248
	YJ5	8	16	47	75	128	213
	YJ6	9	21	54	90	146	265
	YJ7	8	20	54	86	139	249
	YJ8	8	19	48	65	113	193
Longjing	LJ1	9	21	62	97	152	264
	LJ2	9	20	48	66	95	172
	LJ3	9	17	50	69	104	191
	LJ4	9	18	54	78	112	203
	LJ5	9	17	38	57	87	148
	LJ6	9	17	54	88	137	235
	LJ7	8	16	44	58	71	122
	LJ8	8	18	54	81	121	204
	LJ9	9	14	50	79	124	213
	LJ10	9	16	48	73	98	178
	LJ11	7	14	36	42	70	125
Helong	HL1	9	19	56	78	132	216
	HL2	9	18	50	72	110	184
	HL3	9	20	57	82	132	220
	HL4	9	16	49	83	125	215
	HL5	9	18	59	91	146	238
	HL6	9	19	45	68	97	164
	HL7	9	18	57	81	120	207
	HL8	9	19	57	89	127	220
	HL9	8	16	46	68	103	175

Table S6. Taxonomy corresponding to key otu in *Zi-Pi* diagram

ID	taxonomy
botu91164	*Gammaproteobacteria*, *Proteobacteria*
botu11251	*Actinobacteria*, *Actinobacteria*
botu67292	*Alphaproteobacteria, Proteobacteria*
botu54813	KD4-96, *Chloroflexi*
botu11323	*Alphaproteobacteria*, *Proteobacteria*
botu77391	*Actinobacteria*, *Actinobacteria*
botu37802	*Gammaproteobacteria*, *Proteobacteria*
botu46319	*Anaerolineae*, *Chloroflexi*
fotu2107	*Dothideomycetes*, *Ascomycota*
fotu8178	No blast hit

Table S7. Pearson correlation between bacterial communities and physicochemical properties. *, **, and * indicated p values were significant at 0.05, 0.01, and 0.001 level, respectively. EC, electrical conductivity, WSOC, water soluble organic carbon, TN, soluble total nitrogen, TDP, total dissolved phosphorus, NH_4^+-N, ammonium nitrogen, NO_3^--N, nitrate nitrogen**

	EC	clay	silt	sand	pH	WSOC	TN	NH_4^+-N	NO_3^--N	TDP
PCoA1	-0.173	-0.190	-0.486**	0.396*	-0.827**	-0.294	-0.155	-0.094	0.082	-0.203
PCoA2	0.443*	0.094	-0.126	0.040	-0.016	-0.621**	0.372	-0.286	0.491**	-0.041
PCoA3	0.167	-0.358	-0.271	0.330	-0.043	0.177	0.196	0.104	0.106	0.399*

Table S8. Pearson correlation between fungi communities and physicochemical properties. *, **, and * indicated *p* values were significant at 0.05, 0.01, and 0.001 level, respectively. EC, electrical conductivity, WSOC, water soluble organic carbon, TN, soluble total nitrogen, TDP, total dissolved phosphorus, NH_4^+-N, ammonium nitrogen, NO_3^--N, nitrate nitrogen**

	EC	clay	silt	sand	pH	WSOC	TN	NH_4^+-N	NO_3^--N	TDP
PCoA1	0.140	0.176	0.354	-0.304	0.633**	0.279	0.096	0.161	-0.107	0.313
PCoA2	-0.287	-0.299	-0.200	0.259	-0.404*	0.221	-0.238	0.190	-0.242	0.243
PCoA3	0.052	-0.069	-0.010	0.037	-0.069	0.335	0.091	0.089	-0.051	0.371

Table S9. Latitude and longitude of sampling sites

Orchard	Sample	Latitude	Longitude
Yanji	YJ1	42°55′7″	129°33′14″
	YJ2	42°55′8″	129°33′20″
	YJ3	42°55′7″	129°33′21″
	YJ4	42°55′9″	129°33′35″
	YJ5	42°55′10″	129°33′36″
	YJ6	42°55′8″	129°33′32″
	YJ7	42°55′8″	129°33′32″
	YJ8	42°55′11″	129°33′36″
Longjing	LJ1	42°29′46″	129°43′48″
	LJ2	42°29′47″	129°43′47″
	LJ3	42°30′20″	129°43′38″
	LJ4	42°30′18″	129°43′38″
	LJ5	42°30′17″	129°43′39″
	LJ6	42°30′11″	129°43′41″
	LJ7	42°30′49″	129°43′47″
	LJ8	42°30′51″	129°43′47″
	LJ9	42°31′49″	129°43′30″
	LJ10	42°31′51″	129°43′32″
	LJ11	42°32′37″	129°43′25″
Helong	HL1	42°42′46″	129°8′24″
	HL2	42°42′44″	129°8′15″
	HL3	42°42′45″	129°8′20″
	HL4	42°42′42″	129°7′58″
	HL5	42°42′42″	129°8′0″
	HL6	42°42′42″	129°8′0″
	HL7	42°42′37″	129°7′42″
	HL8	42°42′39″	129°7′43″
	HL9	42°42′40″	129°7′47″
